# Influence of age, irradiation and humanization on NSG mouse phenotypes

**DOI:** 10.1242/bio.013201

**Published:** 2015-09-09

**Authors:** Jaclyn S. Knibbe-Hollinger, Natasha R. Fields, Tammy R Chaudoin, Adrian A. Epstein, Edward Makarov, Sidra P. Akhter, Santhi Gorantla, Stephen J. Bonasera, Howard E. Gendelman, Larisa Y. Poluektova

**Affiliations:** 1 Department of Pharmacology and Experimental Neuroscience, University of Nebraska Medical Center, 985880 Nebraska Medical Center, Omaha, NE 68198-5880, USA; 2 Department of Internal Medicine, Geriatrics Division, 986155 Nebraska Medical Center, Omaha, NE 68198-6155, USA

**Keywords:** NSG mice, Hematology, Chemistry, Body mass composition, Mouse home cage monitoring, Time, Locomotion, Behavior, Circadian rhythm

## Abstract

Humanized mice are frequently utilized in bench to bedside therapeutic tests to combat human infectious, cancerous and degenerative diseases. For the fields of hematology-oncology, regenerative medicine, and infectious diseases, the immune deficient mice have been used commonly in basic research efforts. Obstacles in true translational efforts abound, as the relationship between mouse and human cells in disease pathogenesis and therapeutic studies requires lengthy investigations. The interplay between human immunity and mouse biology proves ever more complicated when aging, irradiation, and human immune reconstitution are considered. All can affect a range of biochemical and behavioral functions. To such ends, we show age- and irradiation-dependent influences for the development of macrocytic hyper chromic anemia, myelodysplasia, blood protein reductions and body composition changes. Humanization contributes to hematologic abnormalities. Home cage behavior revealed day and dark cycle locomotion also influenced by human cell reconstitutions. Significant age-related day-to-day variability in movement, feeding and drinking behaviors were observed. We posit that this data serves to enable researchers to better design translational studies in this rapidly emerging field of mouse humanization.

## INTRODUCTION

Non-obese diabetic (NOD) background mice [developed at Shionogi Research Laboratories in Aburahi, Japan (Makino et al., 1980, 1985)] carrying mutation of DNA-dependent protein kinase (*Prkdc*) (Bosma et al., 1983) and deletion (Cao et al., 1995; DiSanto et al., 1995) or truncation (Ikebe et al., 1997; Ohbo et al., 1996; Sugamura et al., 1996) of common cytokine receptor gamma chain (cγ), are termed NOG or NSG mice. The strains are excellent for human stem cells engraftment and T cell reconstitution in the mouse thymus (Ito et al., 2002; Saito et al., 2002). More than 135 studies focused on genetically modified immune deficient mice, which now yield more than 1200 publications and patents (Google Scholar, December 2014). Ten percent of these studies involved stem cell biology, 25% cancer research, 28% immune system development, and 31% therapeutics. The mice have, for example, received significant attention as a preclinical testing platform for human hematopoiesis (Rongvaux et al., 2013; Tanner et al., 2014) and tissue regeneration (Isobe et al., 2014). Studies of human tumor stem cell and tissues biology for cancer research are also operative (Williams et al., 2013). Moreover, human-specific viral infections, such as the human immunodeficiency virus type one (HIV-1), are commonly investigated in such humanized mice and represent 6% of all publications in the field (Sato and Koyanagi, 2011). These include studies of viral pathobiology, innate and adaptive immunity, and therapeutics (Dash et al., 2012, 2011; Gorantla et al., 2012; Nischang et al., 2012). Human mycobacterial (tuberculosis) (Calderon et al., 2013; Lee et al., 2013; Leung et al., 2013) and parasite infections (for example, malaria) (Kaushansky et al., 2014; Vaughan et al., 2012) can also be studied in these mice (Brehm et al., 2014). However, hematologic, metabolic and behavioral characteristics of these animals remain limited. Aging and conditioning regimens for stem cell transplantation, such as low dose irradiation, remain unexplored.

NOD mice, known for their susceptibility to autoimmune insulin dependent diabetes mellitus have multiple genetic abnormalities (Leiter, 1993). The addition of severe combined immune deficiency [associated with point mutation of DNA dependent protein kinase gene (Bosma et al., 1983)], in combination with deletion of common cytokine receptor gamma chain (Cao et al., 1995; DiSanto et al., 1995), abrogates development of lymphocytes and secondary lymphoid structures. A deficiency in the non-homologous DNA end joining process and *scid* mutation also increases sensitivity of stromal, hematopoietic, immune T and B cells, and neuronal progenitors to gamma irradiation (Biedermann et al., 1991; Poluektova et al., 2005). Moreover, genetic modifications and the pre-conditionings required to create humanized mice also lead to inherent limitations in translating data sets from rodents to man (McCafferty et al., 2014). Thus, while capable of long-term stable human cell and tissue engraftments, inherent complexities in the animals themselves can affect their broad utility for human disease testing. Use of such mice to study drug toxicities, pharmacokinetics and metabolism demands robust characterization, especially, in relation to age and animal conditioning regimens. NSG mouse behavior in such a context may be different than normal experimental mice (Rola et al., 2004; York et al., 2012).

To such ends, we report descriptions of hematologic, blood chemistry, and behavioral changes associated with NSG mouse aging by comparisons of 6- and 12-month-old animals. These are referred to as young and middle age, respectively. We observed age-dependent anemia, peripheral blood lymphocytosis and reduced serum albumin. We also describe investigations of each of these parameters reflecting stem cell conditioning such as low dose gamma irradiation performed at birth and the human immune cell reconstitution. Irradiation was shown to affect blood counts and chemistry, body composition and behavior. We posit that such data sets should be considered in the use of these animals for human testing in order to guide final results. This is especially important for therapeutic testing of human-specific viral, bacterial and parasitic infections and of cancer biology.

## RESULTS

### Hematology

Changes in hematologic parameters are a common indication of drug toxicity and inflammation. Applications of aging NSG mice for HIV drug development research require evaluation of consequent low dose irradiation. To this end, we evaluated 6 and 12 month-old NSG and similar aged humanized mice.

#### Red blood cells

**Fig. 1.**
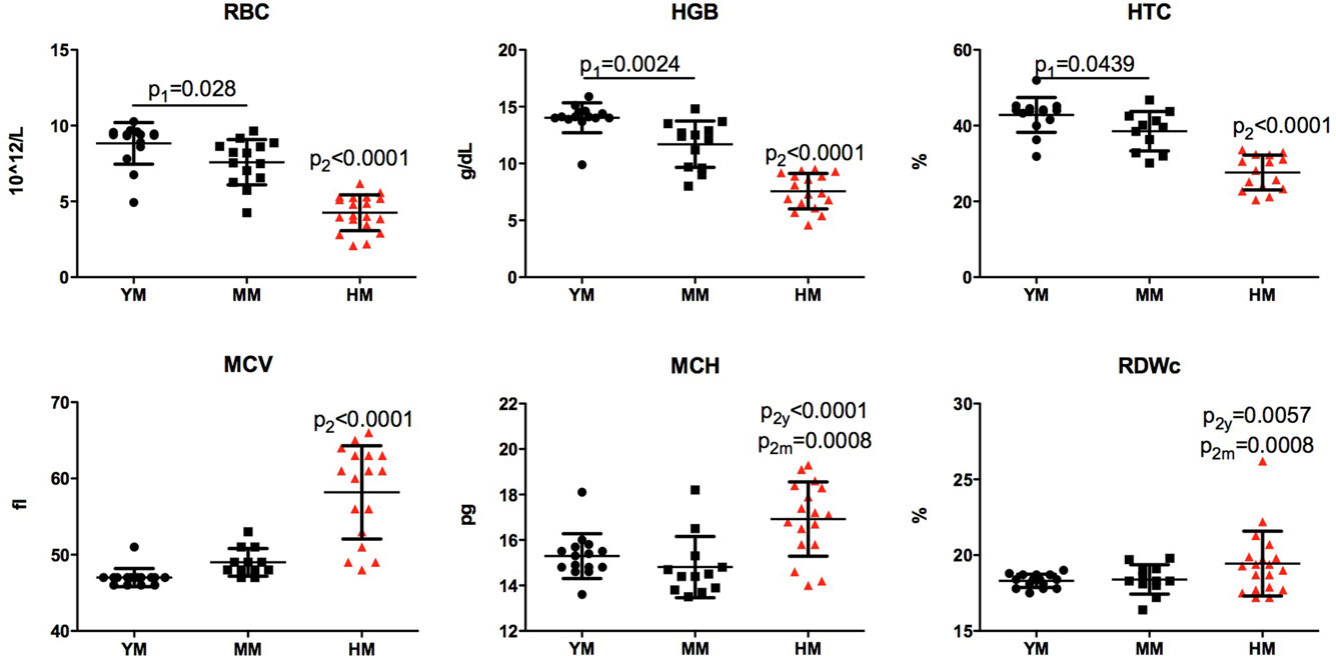
**Age-dependent, irradiation and humanization-induced changes in red blood cells of NSG mice.** Comparison of peripheral blood red cell count (RBC), hemoglobin concentration (HGB), hematocrit (HCT), mean (red) cell volume (MCV), the average amount of hemoglobin (mean corpuscular hemoglobin, MCV) per red blood cell and red cell distribution width (RDWc) between young 6 month old (YM), middle age 12 month old (MM) and humanized (HM) NSG mice. Mild reduction of RBC and HGB levels were found with age and significant changes corresponding to macrocytic hyperchromic anemia were observed in irradiated and transplanted with human hematopoietic stem cells (HSC) NSG mice. One-way ANOVA with post-hoc testing by Tukey's Multiple Comparison Test. Unpaired *t*-test with Welch's correction: p_1_ – differences between YM and MM; p_2_, p_2y_ or p_2m_ – difference between humanized and non-humanized, from YM and MM mice. Individual values, mean and s.d. are shown.

A decline of 15% was observed in red blood cells (RBC) that were associated with age (8.83±0.36 vs 7.59±0.40×10^12^ c/l. *P*<0.05). A more than 50% RBC decrease was seen following irradiation. In humanized mice, RBC mean count was 4.25±0.27×10^12^/l (*P*<0.001). Parallel declines were found for hemoglobin (hgb) and hematocrit (hct) values. These were coincident with increased mean red cell volume (MCV). The average hgb dropped without changes in the distribution of red cell widths (RDW) (Fig. 1, Table 1). Together, these findings demonstrate that irradiation at birth induces a macrocytic hyperchromic anemia, while aging affects a milder anemia consistent with changes observed in aged humans.

**Table 1.**
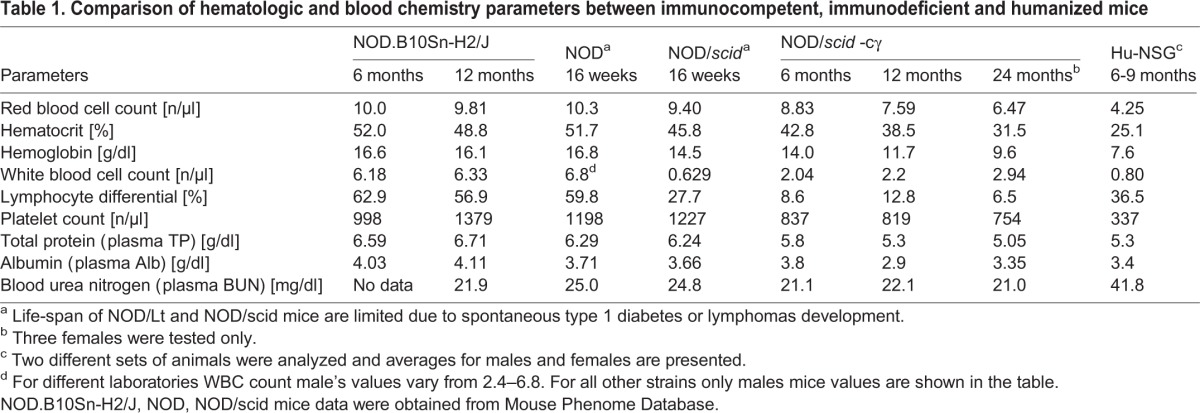
**Comparison of hematologic and blood chemistry parameters between immunocompetent, immunodeficient and humanized mice**

#### White blood cells and platelets

**Fig. 2.**
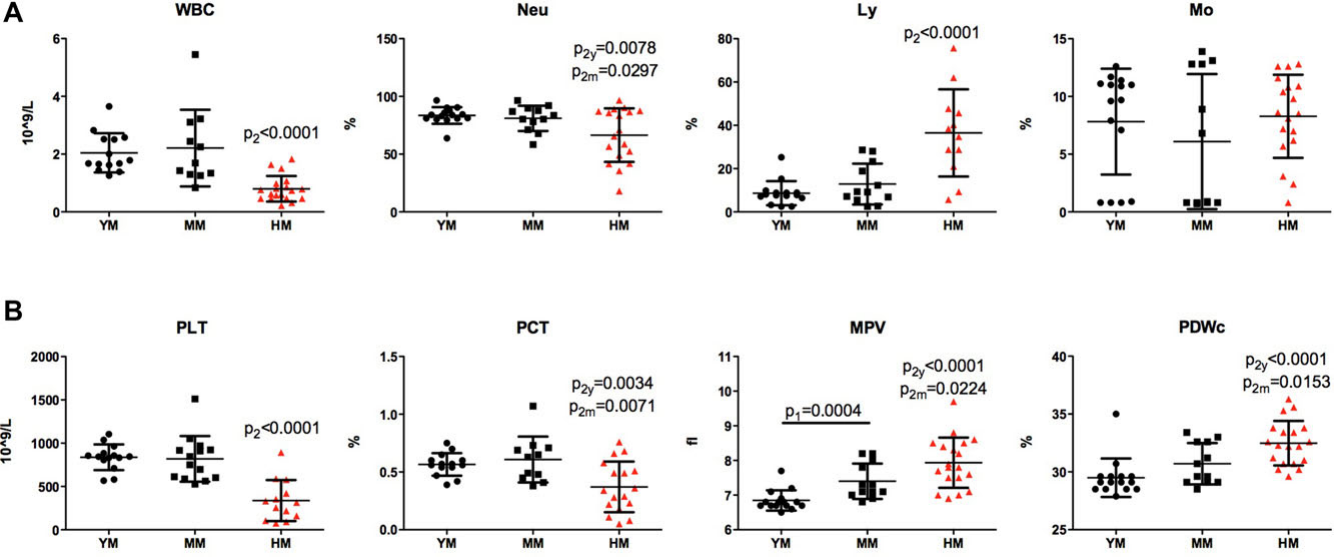
**Irradiation and humanization induced changes in white blood cells and platelets counts of NSG mice.** (A) Comparison of peripheral white cell count (WBC) and differentials (neutrophils, Neu; lymphocytes, Ly; monocytes, Mo) between young 6 month old (YM), middle age 12 month old (MM) and humanized (HM) NSG mice. Significant reduction of WBC and relative decrease in Neu with significant increased Ly percentage in HM NSG mice were observed. (B) Platelet count (PLT) and plateletcrit (PCT), mean platelet volume (MPV) and platelet distribution width (PDWc) are significantly changed in HM with reduction of total number, PCT and elevation of platelet volume and distribution width. One-way ANOVA with post-hoc testing by Tukey's Multiple Comparison Test. Unpaired *t*-test with Welch's correction: p_1_ – differences between YM and MM; p_2_, p_2y_ or p_2m_ – difference between humanized and non-humanized, from YM and MM mice. Individual values, mean and s.d. are shown.

The total number of white blood cells (WBC) also was found to be significantly lower in humanized mice when compared with young and middle-aged NSG mice (2.04±0.18 and 0.80±0.10×10^12^/l, *P*<0.001). Slightly reduced granulocyte numbers (83.5±1.8 and 66.5±5.5%, *P*<0.05) and an increased lymphocyte percentages were found without relative changes in monocytes. These were seen in humanized compared to young and middle-aged NSG mice (Fig. 2A). In immunocompetent mice, over 50% of white cells are lymphocytes (Table 1). Genetically mediated ablation of lymphocytes (*scid* mutation and common cytokine receptor gamma chain deletion) resulted in white cell reductions with significant neutrophil prominence. Humanization of mouse blood by human stem cell transplantation transformed mouse white cell differential counts by increasing the lymphocytes proportions. Young and middle-aged cohorts showed 8.7±1.4 and 12.9±2.7% lymphocytes whereas humanized mice had 36.5±5.8% (*P*<0.001) of such cells. A variable amount of lymphocytes (5-88%) were of human origin.

Similarly, platelet counts were significantly reduced from 837±40 to 338±66×10^12^/l, *P*<0.001 in non- and humanized mice, respectively. However, cell volume and cell distribution were increased (Fig. 2B). In NSG mice, total platelet counts were not changed with age, but mean platelet volume increased. Young and middle-aged mice showed platelet volumes of 6.85±0.08 and 7.40±0.15 fl *P*<0.05 whereas humanized mice had values of 7.94±0.17 *P*<0.001.

#### Correlations between human and murine cell populations

**Fig. 3.**
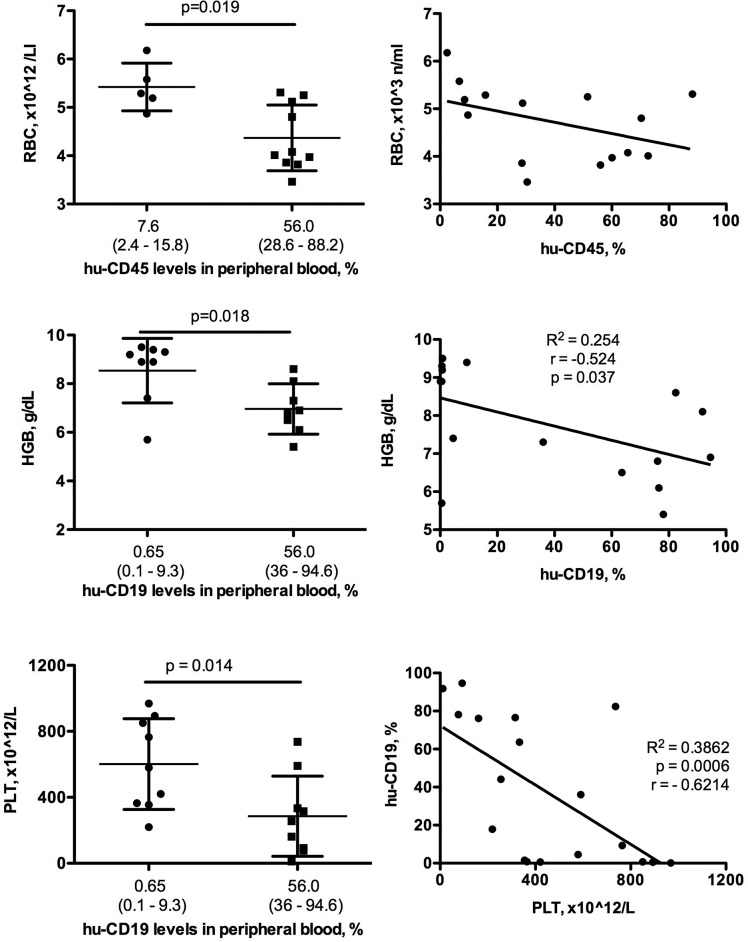
**Comparison of hematologic parameters in humanized mice with different levels of peripheral blood repopulation.** The high levels of humanization detected by the presence of human cells (CD45+) and, specifically, B cells (CD19+) were associated with decreased red blood cell (RBC) count, hemoglobin concentration and platelets numbers (PLT). A non-parametric Mann Whitney test was used to determine statistical significance between groups of mice with varying levels human cell reconstitution. Individual values, mean and s.d. are shown.

The repopulation of mouse bone marrow with xenogeneic human cells was shown to affect mouse hematopoiesis. Also, human adaptive immunity to mouse cells (erythrocytes, for example) can affect mouse hematopoiesis. Thus, correlations were made between the levels of peripheral blood human lymphocyte reconstitution and mouse hematologic parameters. For these tests, animals were reconstituted with human stem cells from different sources including 5 from fetal liver and 11 from cord blood. Significant variabilities of human cells were found in blood. This allowed comparisons between low and high human T and B cell levels with mouse white and red cell and platelet counts. The reduction of red blood cells and platelets in humanized mice was associated with percentages of human CD45+ and B cell numbers. The correlation of B cells with hemoglobin and platelet levels was statistically significant *r*=−0.524 (*P*=0.037, R^2^=0.254) and *r*=−0.621 (*P*=0.0006, R^2^=0.386), respectively (Fig. 3).

### Clinical chemistry

**Fig. 4.**
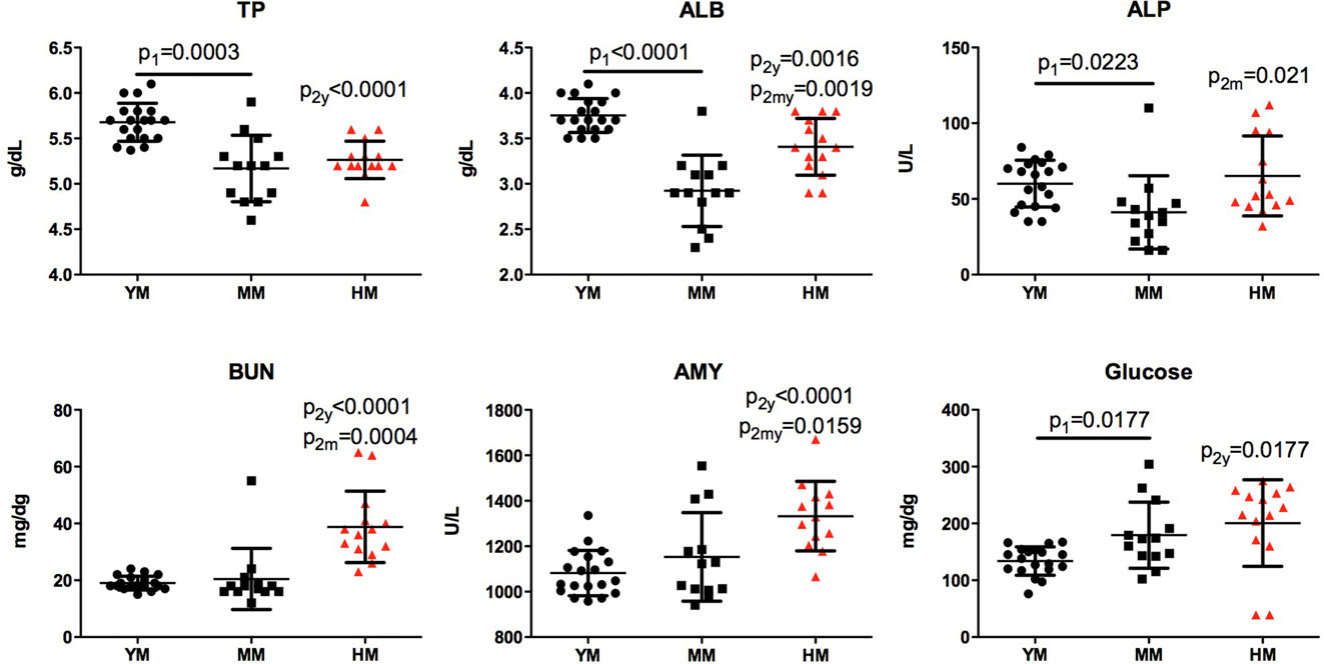
**Age and humanization induced changes in blood biochemistry of NSG mice.** Reduced total protein (TP) and albumin (ALB) concentration in middle age 12 month old (MM) and humanized (HM) NSG mice compared to young 6 month old (YM). Humanization associated increase is shown in alkaline phosphatase (ALP) and amylase (AMY) activity, blood urea nitrogen (BUN) and glucose concentrations. One-way ANOVA with post-hoc testing by Tukey's Multiple Comparison Test. Unpaired *t*-test with Welch's correction: p_1_ – differences between YM and MM; p_2_, p_2y_ or p_2m_ – difference between mice with or without humanization. Individual values, mean and s.d. are shown.

Blood chemistry changes associated with NSG mouse aging, irradiation and humanization were more modest (Fig. 4). Age-dependent and humanization led to a decline in total protein (young 5.7±0.1, middle-aged 5.2±0.1, and humanized 5.3±0.1 g/l, *P*<0.001) as well as albumin levels (young 3.8±0.04, middle age 2.9±0.1, *P*<0.05; and humanized 3.4±0.1 g/l, *P*<0.001). Humanization increased blood urea nitrogen (young 19.0±0.6 and humanized 38.8±3.4 mg/dg, *P*<0.001). However, this change was seen within normal mouse limits. Glucose levels were also within normal limits (125-250 mg/dl). However, increased levels in single housed animals were most linked to the time of blood collection. Repetitive measurements at 0900 (after active eating during night time) and 1600 (resting time) did not show statistically different values.

### Percent lean body and fat tissue mass

DEXA revealed significant differences in tissue-related parameters between humanized mice (HM), young males and females, and middle age males (abbreviated for this section as YM, YF, and MM mice). Humanized mice have less body weight (YM 31.1±3.4 g, HM 20.52±2.59 g; *P*<3×10^−6^), total tissue mass (YM 15.26±1.77 g; HM 9.89±0.83 g; *P*<1×10^−6^), total tissue area (YM 9.95±0.82 cm^2^; HM 7.29±0.47 cm^2^; *P*<1×10^−6^), total bone area (YM 2.77±0.53 cm^2^; HM 2.12±0.14 cm^2^; *P*<0.006), and bone mineral content (YM 0.154±0.03 g/cm; HM 0.111±0.016 g/cm; *P*<0.004), with no difference in bone density, R_ST_, and adiposity. Young mice had less bone mineral density compared with MM mice (YM 0.056±0.005; MM 0.063±0.008 g/cm; *P*<0.027). Young female mice weighed less (YF 26.24±0.87; *P*<0.008) and had less total tissue mass (12.6±1.19 g; *P*<0.009) compared to YM mice. Comparisons between HM versus MM were similar to differences noted between the HM versus YM cohorts (weight MM 30.33 g; *P*<4×10^−6^; total tissue mass MM 15.59±1.39 g; *P*≪1×10^−6^; total tissue area MM 10.14±0.74 cm^2^; *P*≪1×10^−6^; bone mineral content MM 0.17±0.04 g/cm; *P*<0.001; total bone area MM 2.64±0.35 cm^2^; *P*<0.002), with an additional difference in bone mass density (MM 0.06±0.009 g/cm^2^; HM 0.052±0.007 g/cm^2^; *P*<0.02).

### Home cage behavior

**Fig. 5.**
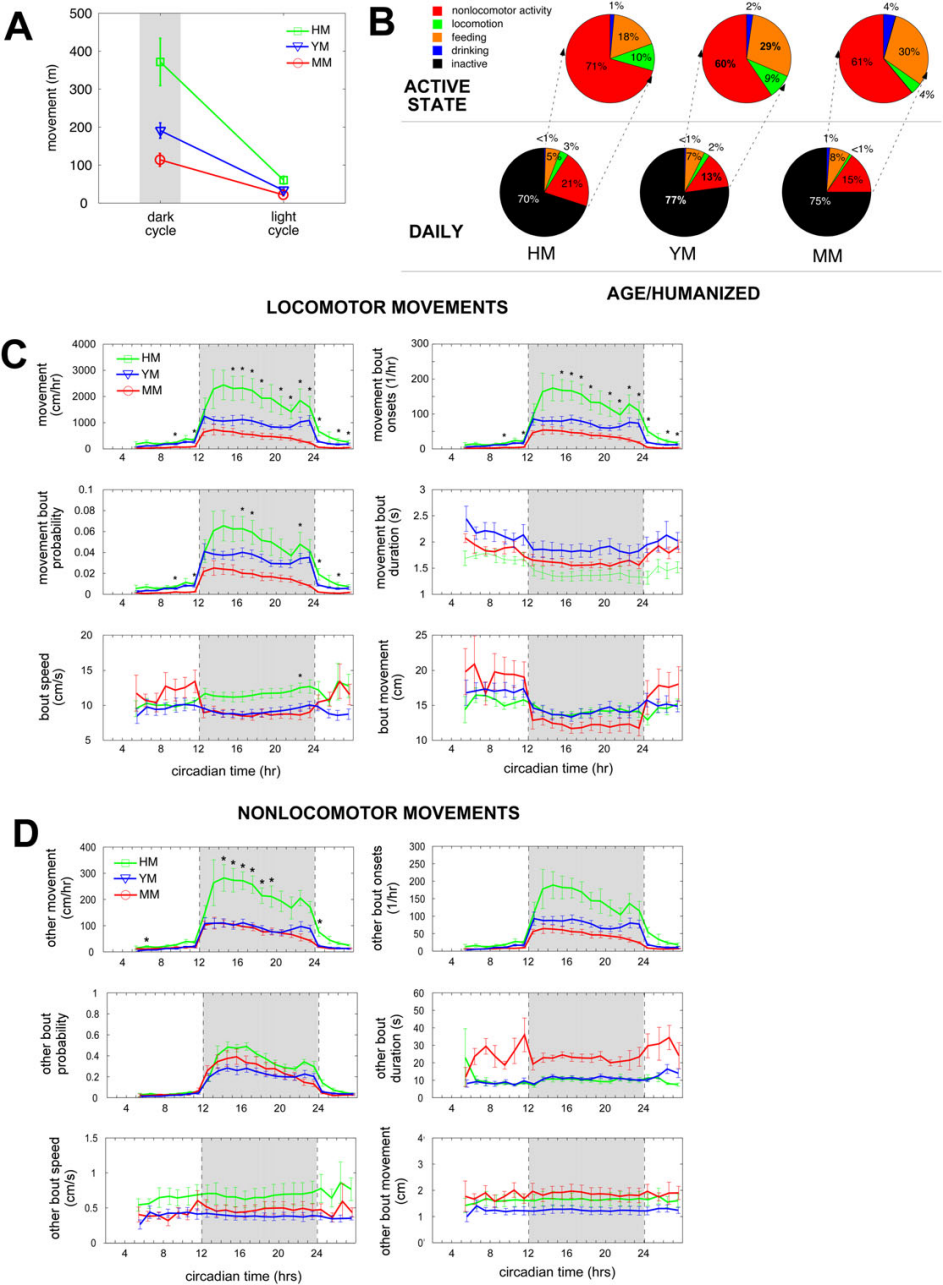
**Mouse home cage behavior monitoring.** Humanized mice (HM) have significantly more dark cycle movement as a result of higher dark cycle movement probability and greater dark cycle movement bouts. (A) Overall movement averages for dark cycle (grayed) and light cycle for humanized, young male (YM), and middle age male (MM) mice (green, blue, red traces respectively). Bars are ±one standard error of the mean. (B) Time budgets show that over 24 h, HM mice have significantly less inactive time compared to YM and MM mice, with a similar increase in nonlocomotor movements (bottom row). Active state time budgets between YM and MM mice are similar except that MM mice show less locomotion compared to YM mice. HM mice active state time budgets are similar to YM, except that HM mice show significantly more nonlocomotor movements, and significantly less time dedicated to feeding, compared to YM mice. (C) Locomotor movement properties. Note that HM mice show increased overall locomotion, locomotor probability, and locomotor bouts compared with either YM or MM mice (asterisks indicate time bins where HM mice significantly differ from YM and MM, Bonferroni corrected for 24 comparisons). (D) Nonlocomotor movement properties. HM mice show increased nonlocomotor movement (albeit one log less than locomotor movements) compared to YM and MM mice. (C,D) Bars are ±one standard error of the mean. No other properties demonstrated statistically significant differences. Overall, these data show that HM mice have increased dark cycle locomotion compared to YM and MM mice, and that this increase is a result of HM mice spending less time in the inactive state, and having greater locomotor probability and number of locomotor bouts (as well as greater overall nonlocomotor movement and a trend toward increased nonlocomotor bouts).

There were 16 observation days for all mice. We chose the young male group as the ‘control’ against which all other groups were compared. Using our FDR criteria, we found 62/565 observed behaviors differed between young and middle-aged males; 50/565 observed behaviors differed between young females and middle-aged males; 168/565 observed behaviors differed between humanized and middle-aged males; 1/565 observed behaviors differed between young males and young females; 1/565 observed behaviors differed between humanized males and young females, and no observed behaviors differed between humanized males and young males (supplementary material Tables S1-S6). In all cohort comparisons, behaviors related to movement (including total locomotion, dark cycle and light cycle locomotion, bout patterns of locomotion, and locomotor time budgets) were over represented. We also noted over representation of feeding-related behaviors in the young versus middle age males. Thus, if 3 and 7 behaviors are falsely present in the young male versus middle age male and humanized male versus middle age male comparisons (respectively), the above inferences regarding differential observed behaviors remain robust (Fig. 5).

#### Irradiation with HSC reconstitution and aging significantly impact mouse movement

At the most coarse level of discrimination, there were marked differences in total daily movement, dark cycle (DC) movement and light cycle (LC) movement between the middle-aged male and humanized male groups (Fig. 5A; for daily, DC, and LC movement; FDR-adjusted *P*<0.02, 0.02, 0.01, respectively). Comparisons of young male versus middle-aged male groups and young male versus humanized male groups were not significant by FDR. Overall, movement is greatest for humanized mice, and least for aged, non-humanized mice.

These gross changes in movement are further reflected in the overall 24-h time budgets of young male, middle-aged male, and humanized male mice, and the sub-budgets quantifying behaviors performed during mouse active states. For time budgets over the entire day, aging was associated with less locomotion (daily, DC, and LC; *P*<0.01, 0.02, 0.01, respectively) and more episodes of stopping and starting (daily, DC, and LC; *P*<0.01, 0.02, not different, respectively). By contrast, humanization increased daily time budget for locomotion (daily, DC, and LC; *P*<0.02, 0.02, 0.01, respectively) and the number of starting/stopping episodes (total, DC, and LC; *P*<0.03, 0.05, 0.02, respectively). Within the active state time budget, aging again decreased total percent time spent in locomotion (daily, DC, and LC; *P*<0.002, 0.0005, not different, respectively), with no effect on feeding, drinking, and other movements. As was the case for the 24-h time budget, the active state time budget of humanized mice showed significant increases in locomotion (daily, DC, and LC; *P*<0.01, 0.01, not different, respectively), and more stopping/starting episodes (daily, DC, and LC; *P*<0.02, not different, 0.01, respectively). Thus, aging decreases the percent of each day (and the percent of total active time) that NSG mice spend in locomotion, while humanization has an opposite effect.

Temporal patterns of locomotion and other movement bouts provide insight into the underlying systems altered by both aging and humanization. For locomotion (Fig. 5C), humanized male mice have a greater number of locomotor bout onsets per hour (*P*<0.02; DC and LC), higher bout speed (*P*<0.02; DC and LC), shorter bout duration (*P*<0.02; DC and LC), and greater total bout movement (*P*<0.01; DC and LC) while moving the same total distance as young male mice. Similarly, humanized male mice cover significantly greater distance in other movement bouts (Fig. 5D) compared to young male mice over both the dark and light cycles (*P*<0.01 DC, *P*<0.01 LC), with similar trends in movement bout duration (*P*<0.02 DC, *P*<0.01 LC), speed (*P*<0.02 DC, not different LC), and onsets (*P*<0.01 DC, *P*<0.01 LC). These factors suggest that CNS systems governing movement bout probability drive an increased number of bout onsets per hour, ultimately leading to increased locomotion in humanized male mice compared to the young males.

Similar findings explain movement differences between young male and middle-aged male cohorts. Young male mice have more bout onsets per hour (*P*<0.01), longer bout duration (*P*<0.01), and similar locomotor speeds (Fig. 5C). Again, the increased probability of locomotion in young male mice drives the increased number of observed locomotor bouts, ultimately accounting for increased locomotion in young male versus middle-aged male mice. Of note, patterns of nonlocomotor movements in young male versus middle-aged male mice are not similar to those observed in young male versus humanized male mice (Fig. 5D). While young males had more hourly nonlocomotor movement bouts compared to middle-aged males, the middle-aged males nonlocomotor movement probability and bout speed are the same as young male, and middle-aged bout duration and total movement per bout are greater than that observed for young males. Thus, middle-aged males appear to compensate for fewer nonlocomotor movement bouts by increasing bout duration, and thus covering more ground per nonlocomotor movement bout compared to young males.

#### Day-to-day correspondence of activity and drinking is greater in humanized mice and less in aged males

**Fig. 6.**
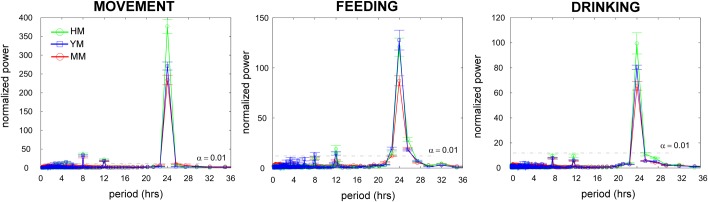
**Day-to-day correspondence of activity.** Humanized male mice (HM) have the least day-to-day variance in patterns of movement, feeding, and drinking. Lomb-Scargle periodograms demonstrate significant periodicities (peaks above the α=0.01 line), as well as group differences between HM, young male (YM), and middle age (MM) mice (green, blue, red traces respectively). Bars are ±one standard error of the mean.

In general, we observed few differences in parameters describing the circadian properties of activity, movement, feeding, and drinking between all mouse cohorts. We noted no differences in average activity onset times, activity offset times, or overall active phase duration. Periodicity analysis examined overall patterns of movement, feeding, and drinking identified significant circadian differences between humanized and the other mouse cohorts. Specifically, the area under the curve for the 24-h periodicity was greater in humanized males (for movement and drinking) compared to other cohorts. This finding suggests that the autocorrelations over 24 h windows for humanized males movement and feeding are stronger than those observed for the other mouse cohorts. Lomb-Scargle periodograms also reveal that the overall area of the 24-h activity peak is greater for young compared to aged mice for movement, feeding, and drinking. This finding has been observed in comparisons between young and aged C57BL/6, BALB, and CBA mouse strains (our unpublished data). This finding suggests that mouse autocorrelations (over 24 h) in movement, feeding, and drinking behaviors are age affected (Fig. 6).

## DISCUSSION

The generation of humanized mice requires detailed descriptions of the genotype, age, sex, and conditioning regimens. Humanization itself shows animal-to-animal variance as does time following any procedure as well as the sources of the cells. Complicating testing even further are the variances of the microbial pathogens studied, which includes viruses, parasites and bacteria. In the quest to use such animals for biomarker and extended therapeutic studies of microbial susceptibility, graft longevity and anti-viral/microbial drug resistance all become notable considerations (Choudhary et al., 2009; Nischang et al., 2012). If such factors were not complicated enough, hematologic abnormalities and altered body weight seen in humanized mice serve to complicate studies even further. The reduction of body weight by low dose irradiation is linked to reduced bone density but to increased fat tissue proportion. Multiple genetic defects present in NSG mice affects multiple cell/organ functions (Kikutani and Makino, 1992; Makino et al., 1985; Shultz et al., 1995) whose description can be found on the Jackson Laboratories web site (http://jaxmice.jax.org/strain/005557.html). The most unexplored parameter for NSG mice is behavior. To this end, we have presented a detailed analysis of age-dependent NSG mouse behavior together with the effects of low dose irradiation and humanization.

We found an age-dependent deficiency in erythropoiesis that began by age 12 months. Reductions in red cell counts, hematocrit and hemoglobin concentrations suggested a more significant defect in hematopoiesis and beyond the *scid* mutation (Qing et al., 2012). The absence of lymphotropic cytokine signaling by the common cytokine receptor gamma chain and the near absence of lymphoid lineage B, NK and T cells could be indirectly involved in the erythrocyte effect. Alternatively, bone marrow derived macrophages can suppress erythroid lineage development and change properties. This could be considered an age-related myelodysplasia. Moreover, hematologic abnormalities can be linked also to irradiation and humanization. The levels of human cell repopulation of mouse lymphoid organs correlate with the proportion of blood lymphocytes. The decline of red blood cells and platelets are also associated with the proportion of human B cells. Human immune cells transplanted into immunodeficient mice may also be able to produce anti-erythrocytes antibodies as a consequence of graft-versus-host disease (Pirruccello et al., 1992). This suggests that immune components could contribute to the observed anemia. However, in the humanized mice used in this report such high concentrations were never achieved despite the observed anemia (Greenblatt et al., 2012). Aged NSG and low-dose irradiated animals could be more sensitive to myelosuppressive drugs. Other drugs may perhaps increase the numbers of red cells and platelets. Humanized mice could, in this manner, serve as a model to study graft-versus-host disease (Bruck et al., 2013; Covassin et al., 2013; Greenblatt et al., 2012).

Among tested blood biochemistry parameters, age-dependent total protein and albumin levels were also reduced. Serum albumin levels are already known to decrease with age in several inbred strains. This could be associated with increased loss due to proteinuria and kidney disease, accelerated catabolism, liver inflammation and increased endotoxin sensitivities (Quimby and Luong, 2007). Interestingly, none of such pathologic conditions were reported previously for NSG mice.

NSG feeding, drinking, and locomotor phenotypes arising from combined *Prkdc* and IL2R-γ chain genetic lesions on a NOD genetic background were examined by mouse home cage behavior tests. Multiple locomotor phenotypes were observed. First, middle-aged male NSG mice have less total locomotion and fewer movement bouts over the circadian day compared to young controls (we anecdotally observe that young NSG mice have less total locomotion and fewer locomotor bouts compared to young male C57BL/6 and BALB mice; data courtesy E.H. Goulding, Northwestern University). Similarly, aging NSG mice show reduced nonlocomotor activity, and fewer nonlocomotor activity bouts compared to young controls. Aged NSG mice spend a smaller percentage of their day and during their total activity time in locomotion compared to young NSG mice. The opposite phenotype is observed when humanized NSG mice are compared to young controls. There are, to date, no published reports of NOD (or other strains of Swiss-Webster superfamily), *Prkdc*, or IL2R-γ baseline locomotor function, complicating interpretation of this locomotor phenotype. However, in related assays measuring open field locomotion (albeit over a significantly shorter time period), NOD mice routinely demonstrate *greater* total locomotion compared to other strains (Amrani et al., 1994; Bothe et al., 2005; Miller et al., 2010). *Prkdc* knockout mice have no genotypic differences in open field locomotion (Brynskikh et al., 2008); there are no published reports of IL2R-γ mice open field locomotion (described as ‘languid’, Garrelds et al., 2002). These results demonstrate that NSG mice that have not undergone immune reconstitution appear to have a baseline locomotor deficit that worsens with age.

We see a dramatic increase in circadian and total locomotor and nonlocomotor movement in NSG mice after human immune reconstitution. This phenotype, resulting from increased dark cycle locomotor bout frequency represents an immune effect on action selection at the basal ganglia. Such neural commands to increase locomotor bout frequency converge on the striatum (Grillner et al., 2005; Hauber, 1998), and evoke facilitation of direct pathway through medium spiny neuron D_1_ receptors as well as inhibition of indirect pathway through medium spiny neuron D_2_ receptor signaling (Kravitz et al., 2010). Our findings suggest that basal ganglia action selection (Bogacz and Gurney, 2007; Gurney et al., 2001) is affected by immune reconstitution. Immune reconstitution of NSG mice decreased, whereas aging increased day-to-day temporal movement variability, feeding, and drinking when compared to controls. We routinely observe significant age-related increases in movement, feeding, and drinking variability across all inbred strains tested to date (S.J.B., unpublished data). Increased behavioral variability with age may be attributed to overall age-related changes in hypothalamic signaling pathways, including those converging upon NFκB (Jiang et al., 2001; Zhang et al., 2013). Potential mechanisms underlying this circadian dysregulation include altered *NPAS2* function (Hoffman et al., 2008). Cytokines have well-established effects on hypothalamic function (Coogan and Wyse, 2008; Turnbull and Rivier, 1999); the altered immune status of both naïve and humanized NSG mice may thus contribute to circadian dysregulation.

Regarding other behaviors, NOD mice showed reduced mean hindpaw withdrawal latencies when compared with non-diabetic strains; NOD mice were also abnormal in their general appearance, activity level, posture, gait and muscle bulk. These findings raise the possibility that mice arising from NOD strains have primary impairments within sensory pathways (Davar et al., 1995). NOD background on behavior testing in several laboratories showed different degrees of increased vertical activity and wildness (Bothe et al., 2005; Wahlsten et al., 2003). Sheltering behavior and locomotor activity of NOD strain in home cage behavior testing of young mice also showed differences with C57bl and Balb/c mice (Loos et al., 2014). Common cytokine receptor chain knockout and *scid* mutation also increased vulnerability of mouse neurons and behavior abnormalities were reported (Brynskikh et al., 2008; Petitto et al., 1999; Vemuri et al., 2001). In addition to these genetic changes is the low dose irradiation known to have a profound effect on mouse neurobehavior physiology (Hayashi et al., 2008; Ko et al., 2009). In comparison to most studies, Balb/c or C57Bl/6 mice, NOD background has been associated with behavior “strangeness”. However, signs of behavior aging of NSG mice similar to these two strains and associated with reduced locomotion, increased episodes of stopping and starting, longer duration of food intake bouts were observed. Because of the parallel appearance of both behavioral and actuarial aging, the slope of such changes would be helpful investigate in the direct comparison to other often used strains for behavior studies. Moreover, accelerated behavior aging of NSG mice, if it is real, could be directly attributed to the severe immunodeficiency and increased incidence of inflammatory reaction such as glial activation observed in NSG mice (Gorantla et al., 2010).

A combination of NOD background, immune deficiency and humanization can lead to hyperactivity. Irradiated NSG had significantly increased motor activity as observed in the home cage monitoring. This finding is different to reported decreased activity in home cage of one-year-old C57Bl/6 mice irradiated at 2 weeks of age (Kalm et al., 2013). This discrepancy could be related to the time of irradiation of one-day old animals which most probably retained a greater ability to compensate damage of low dose irradiation of 1 Gy than two-week-old mice irradiated at 6 Gy. Overall, the effect of irradiation at different ages on volumes of the brain subregions and inhibitory regulation networks are noted. The relationships between brain structural volume and motor performance measures are possible and widely accepted (Seidler et al., 2010). For example, primary motor cortex atrophy, decline in white matter and subcortical structures, substantia nigra volumes may contribute to the movement slowing seen with age. Taken together, the impact of age and irradiation for neuromotor function on the NSG mice are noteworthy. Pattern alterations for neural plasticity are age dependent. Drug testing and application of NSG mice for specific behavior studies such as decreased abilities to engage relevant neural circuitry, activation and anxiety likely reflect changes in sensory information processing.

## MATERIAL AND METHODS

### Mice

All manipulations with mice were approved by University of Nebraska Medical Center Institutional Animal Care and Use Committee (IACUC). The diet provided is Teklad LM-485 (7012), which is autoclaved for sterility. The mice are housed in a sterile environment and only accessed under a BSL2 hood. The mice are monitored daily for health, food, and water, and cages are changed biweekly. We examined a cohort of young females (6 month old; *n*=7) and males (*n*=8), aged females (12 month old; *n*=4) and males (*n*=8), and humanized mice (6 and 9 month old; *n*=19). Breeding and normal mice collection was done according to appropriate protocols (Yuan et al., 2011).

### Humanized mice preparation

Humanized mice generation begins with irradiation of pups after the first or second day of birth with a 55 s exposure equaling 1.1 Gy using X-ray irradiator (Rad Source RS-2000 Biological System). Four hours after irradiation, pups are injected intrahepatically with CD34+ human derived stem cells. Depending upon the source, 500,000 fetal liver cells or 100,000 cord blood cells are injected per mouse. The humanized mice used in this study are identified for which cells were used. Pups are weaned at 4 weeks of age, and are evaluated starting at 9 weeks for human cell markers (CD45, CD3, CD8, CD4, CD19, CD14) via flow cytometry analysis (BD FACS Diva). We examined young 6-month-old females (*n*=5) and males (*n*=2), middle age 9-month-old females (*n*=3) and males (*n*=9). End-point FACS data of human cells populations in peripheral blood were used for correlation analysis.

### Complete blood count and blood and urine chemistry

Complete blood count (CBC) was analyzed with the Abaxis VetScan HM5 hematology machine. Blood was collected in potassium EDTA microtainer tubes (BD 365973) via cheek bleed or heart puncture. Approximately 20–30 μl of blood was used and kept at room temperature for less than 2 h to avoid hemolysis.

Chemistry analysis was measured with the Abaxis VetScan VS2 chemistry machine. The comprehensive panel (DVM Resources 106144) includes albumin (ALB), alkaline phosphatase (ALP), alanine transaminase (ALT), amylase (AMY), total bilirubin (TBil), blood urea nitrogen (BUN), calcium (CA+), phosphate (PHOS), creatine (CRE), Glucose, NA+, K+, total protein (TP), and globulin (Glob). Approximately 100 μl of blood was collected via cheek bleed or heart puncture in lithium heparin tubes (BD 365965). Samples were kept and read at room temperature up to 4 h to avoid hemolysis. Urine glucose and protein analysis was done with Chemstrip 2 GP (Roche-Cobas; #11895397160). Statistical analysis for CBC and blood chemistry was done by one-way ANOVA Bartlett's test for equal variances and Tukey's Multiple Comparison Test. Pearson's correlation and Mann–Whitney test were applied for analysis of humanized mice with different levels of reconstitution (GraphPad Prism 5).

### Mouse home cage monitoring

Details regarding home cage monitoring (HCM) system design and data analysis algorithms have been described (Goulding et al., 2008; Parkison et al., 2012). Briefly, mice are individually housed in low profile (48.3 cm long×25.7 cm wide×15.2 cm high, PC10196HT, Allentown) instrumented cages with *ad lib* access to food (while breaking a photobeam) and water (while activating a capacitive lickometer). Mouse position is determined by solving exact equations of torque measured by load cells (LSB200, FUTEK) positioned at the left and right cage front corners and the center of the cage back. All instruments are sampled at 1 kHz; data is written to binary form using LabView software controlling a real-time computer (National Instruments, TX, USA) and subsequently processed using custom written MATLAB (MathWorks, MA, USA) code. Mice are fed powdered chow (PicoLab Mouse Irradiated, 5058, OH, USA) and autoclaved water (prepared in house). Facility lighting was controlled by the UNMC Comparative Medicine Department on a 12-h-on/12-h-off (0600 to 1800 military time) period for this experiment. Our system lightmeter (Li-Cor LI-210SA, with 2290MV adapter/LI-190/191/210) confirms near absence of light during dark cycle (meter voltage indistinguishable from RMS noise centered at 0 mV). Peak illuminance during light cycle is approximately 1270 lux. Facility temperature ranged between 22.8 and 24.4°C; relative humidity ranged between 5 and 40%.

Our HCM system is housed in a soundproofed room that completely blocks out noise from the animal facility; access is key-card limited to two investigators (SJB, TRC). We examined cohorts of young male NSG (6 month old; *n*=8), young female NSG (6 month old; *n*=7), aged male NSG (12 month old; *n*=10, and young male “humanized” NSG mice after total bone marrow ablation and reconstitution with human marrow stem cell precursors (6 month old; *n*=7). Our mouse home cage monitoring system measures hundreds of distinct behaviors after quality control and classification from 6 primary data sources (four analog corresponding to voltage from three load cells and one lightmeter; two TTL corresponding to off/on state of photobeam and licking sensors). All cohorts were continuously observed (except for very brief periods daily to check mouse status and every 3–4 days to replenish water and food supplies) for 21 days (including 5 day habituation period).

#### Statistics

Mouse periodicities were determined using the Lomb-Scargle algorithm (Scargle, 1982); this approach provides robust estimates of behavioral periods despite unevenly sampled data. Mouse bout criteria were determined by fitting a Gaussian mixture model to the feeding and drinking inter-event intervals (temporal criteria, Tolkamp et al., 1998; Tolkamp and Kyriazakis, 1999), and by examining the maximum distance that the mouse moved from either the feeder or lick spout between consecutive feeding or drinking events (spatial criteria). Exceeding either the temporal or spatial criteria for remaining within a given bout led to termination of current bout and start of subsequent bout. Movement bouts were determined by examining the speed and turn angle characteristics of mice engaged in pure locomotion; these properties were used to classify all movements within the system. Full details of classification are provided in the supplementary data of Goulding et al. (2008). Given the large number of behavioral measures contained within our datasets, we first controlled familywise error rates across all measures except periodicity. Unless stated otherwise, we used a false discovery rate (FDR; calculated per MAFDR, MATLAB) statistic set at *P*<0.05 (Benjamini and Hochberg, 1995; Storey, 2002); this same approach is well-accepted to manage familywise error rates in gene expression experiments involving >10,000 potential comparisons.

### Dual energy x-ray absorptiometry (DEXA)

Cohorts and cohort sizes as described for mouse home cage monitoring. Mice were briefly anesthetized with isoflurane (1.5–2.0 vol %) until unresponsive to tail pinch. Mice were then placed on the DEXA stage (PIXImus, GE Lunar) for imaging. Values for bone mineral density (BMD), bone mineral content (BMC), bone area (B area), tissue area (T area), ratio of soft tissue attenuation for two photon energies (R_ST_), percent fat, and total tissue mass (TTM) were calculated using LUNAR PIXImus 2.10. Mice were weighed with a digital scale (SP202US, OHaus), and then returned to their housing cage for recovery. All mice tolerated the procedure well, and no complications were noted. DEXA data were analyzed by 2-sided Student's *t*-test, Bonferroni corrected for 8 comparisons.
